# Keep calm and exercise on: A single bout of exercise supports executive function across a continuum of trait anxiety levels

**DOI:** 10.1016/j.xjmad.2026.100200

**Published:** 2026-08-29

**Authors:** Lian Buwadi, Gianna Jeyarajan, Alma Rahimi, Matthew Heath, Benjamin Tari

**Affiliations:** a Graduate Program in Neuroscience, University of Western Ontario, 1151 Richmond St., London, ON N6A 3K7, Canada; b School of Kinesiology, Faculty of Health Sciences, University of Western Ontario, 1151 Richmond St., London, ON N6A 3K7, Canada; c Canadian Centre for Activity and Aging, University of Western Ontario, 1201 Western Rd., London, ON N6G 1H1, Canada; d Institute of Sport, Exercise and Health, University College London, 170 Tottenham Court Rd., London W1T 7HA, United Kingdom

**Keywords:** Aerobic exercise, Antisaccades, Inhibitory control, Oculomotor, State-Trait Anxiety Inventory

## Abstract

Executive function (EF) is a cognitive construct supporting activities of daily living. Trait anxiety (TA) is a stable predisposition to experiencing anxiety that some work has linked to an EF deficit. In turn, a single bout of aerobic exercise elicits a postexercise EF benefit and therefore may serve as an intervention to ameliorate TA-related EF deficits. Here, we examined whether postexercise EF benefits differ across individuals along the continuum of TA. Participants (N = 31) completed a single 25-min bout of moderate-intensity aerobic exercise (via cycle ergometer) and an equivalent duration non-exercise control condition. TA was assessed via the State-Trait Anxiety Inventory (STAI) and pre- and post-condition EF was assessed via the antisaccade task. STAI values ranged from 23 to 60 and produced an average of 42 (SD = 10), and a linear mixed model employing STAI as a covariate showed that the variable was positively correlated with reaction time (RT) across all experimental conditions. Moreover, a higher-order interaction showed that the exercise condition – but not the control condition – produced a pre- to post-condition reduction in antisaccade RTs and was a result that did not vary across the continuum of participant-specific STAI values. Thus, although increased TA was associated with reduced information processing efficiency, TA status did not impact the extent to which exercise provided an EF “boost”. Accordingly, single bout and prospective chronic exercise programs may serve to ameliorate TA-based EF changes.

## Highlights

• Trait anxiety (TA) is a bias to anxiety linked to executive function (EF) deficits.
• We compared baseline and postexercise EF benefits across a TA spectrum.
• A postexercise EF benefit was observed across the TA spectrum.
• Exercise may serve as a viable tool to benefit EF in persons with low- and high-TA.

## Graphical Abstract

**Figure:**
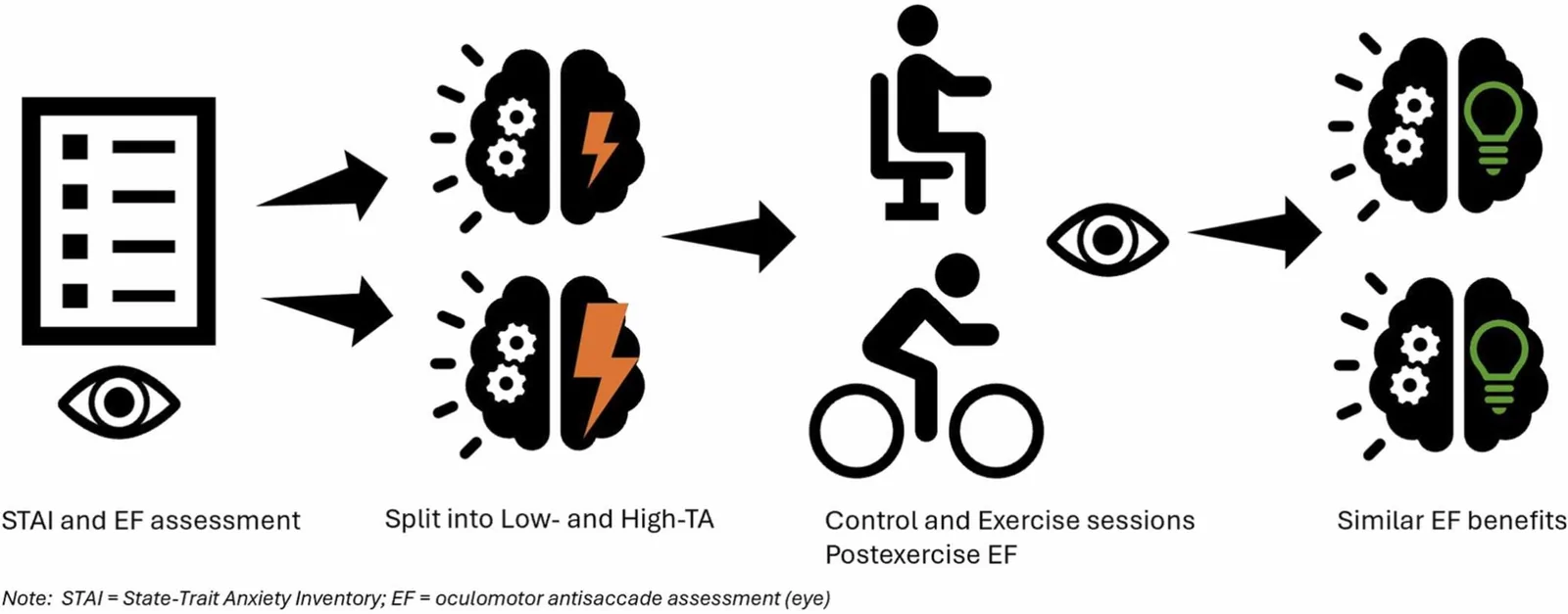

## 1 Introduction

Executive function (EF) includes the core components of inhibitory control, working memory, and cognitive flexibility and is a cognitive construct essential for activities of daily living [1], [2]. Given the importance of EF, a salient goal is to identify psychological and physiological states that negatively impact EF and to develop/identify interventions that support EF. Trait anxiety (TA) is a stable personality dimension wherein an individual demonstrates a tendency to experience and report negative emotions such as fear, worry, and anxiety across a myriad of daily situations [3]. Individuals with high levels of TA are more likely to perceive everyday situations as stressful and are at greater risk for anxiety-related disorders including depression and impaired social functioning [4], [5]. As well, there is some evidence that persons with high TA exhibit impairments in EF, and neuroimaging work has linked this impairment to task-based activity within dedicated EF networks that is distinct from persons with low TA [6], [7], [8].

A TA-based EF impairment has been linked to an overreliance on stimulus-driven and bottom-up processing that prevents an individual from evaluating a current state/goal via top-down cognitive control [9]. In particular, the attentional control theory (ACT) states that persons with high-TA perform like persons with low-TA in terms of EF task accuracy (i.e., performance effectiveness); however, maintained accuracy is associated with the ‘cost’ of increased processing time (i.e., performance efficiency). In support of the ACT, Ansari and Derakshan used the State-Trait Anxiety Inventory (STAI) to recruit participants with low- (STAI < 34) or high-TA (STAI > 44) in advance of an oculomotor assessment of EF (i.e., pro- and antisaccade) [6]. Prosaccades are a goal-directed eye movement (i.e., saccade) requiring a response to the veridical location of an exogenously presented target and are mediated largely independent of top-down EF via retinotopic projections to the superior colliculus [10]. In contrast, antisaccades require a response mirror-symmetrical to a target and result in longer reaction times (RT) [11], increased directional errors [12] and less accurate and more variable endpoints [13], [14] than prosaccades. Behavioural, clinical and neuroimaging work in humans, as well as single-cell recording and transient cooling studies in non-human primates, has attributed antisaccade behavioural ‘costs’ to the EF demands of suppressing a pre-potent prosaccade (i.e., inhibitory control) and the 180º spatial transposition of a target’s coordinates (i.e., vector inversion) [15]. Ansari and Derakshan reported that persons with high-TA exhibited longer antisaccade – but not prosaccade – RTs compared to their low-TA counterparts, whereas both groups showed comparable directional errors (i.e., a prosaccade instead of an instructed antisaccade and *vice versa*). Similarly, Wong and colleagues classified participants into low- (STAI ≤ 37) and high-TA (STAI ≥ 38) groups and examined their performance on a parametric go/no-go inhibitory control task [16]. Results showed that individuals in both groups exhibited comparable response accuracy; however, the high-TA group exhibited longer RTs compared to their low-TA counterparts and the magnitude of the difference increased with increasing task difficulty. Moreover, Basten et al.’s fMRI findings showed a positive linear relationship between STAI scores and task-based activity in the dorsolateral prefrontal cortex (DLPFC) during incongruent Stroop trials (i.e., a measure of inhibitory control) and concluded that such a result evinced decreased neural processing efficiency in persons with high TA [7]. Thus, convergent evidence suggests that persons with high-TA devote increased processing resources to an EF task to maintain an optimal performance effectiveness.

The present work sought to determine whether a putative decrease in EF efficiency with increasing TA is ameliorated following a single bout of aerobic exercise. An exercise intervention was selected because of extensive evidence showing that single bouts of aerobic and/or resistance exercise provide a postexercise EF benefit in healthy young and older adults and individuals with a range of neurodegenerative (e.g., Alzheimer’s disease, mild cognitive impairment, Parkinson’s disease) and neuropsychiatric (e.g., major depression disorder, substance use disorder, schizophrenia) conditions [17], [18], [19]. Further, a postexercise EF benefit is observed for low- to high-fit individuals [20] and across a continuum of metabolically sustainable exercise intensities (i.e., light to heavy intensity) [21], [22] and persists for up to 60-min [23], [24], [25], [26]. For example, Heath et al. (2018) and Petrella et al. (2019) had healthy young and older adults, respectively, complete 10-min single bouts of aerobic exercise in moderate (i.e., 80% of lactate threshold), heavy (i.e., 15% of the difference between V̇O_2_ peak and lactate threshold) and very-heavy (i.e., 50% of the difference between V̇O_2_ peak and lactate threshold) exercise conditions and observed a 7% pre- to postexercise reduction in antisaccade – but not prosaccade – RTs. The postexercise EF benefit has been linked to interdependent exercise-based increases in biomolecule levels (e.g., brain-derived neurotrophic factor, catecholamines) [27], [28], cerebral blood flow [29], [30] and functional connectivity [31] that elicits thermo-mechanical changes improving EF network processing efficiency *and* effectiveness.

To our knowledge, no studies have directly examined whether a single bout of aerobic exercise differentially impacts a postexercise EF benefit across a continuum of TA levels. This is a salient question given that exercise may serve as a simple, accessible and cost-effective means by which persons with increased TA may accrue improved EF in advance of educational-, sport-, and occupation-critical events. In the present work, participants completed the STAI and on separate days completed 25-min of moderate intensity aerobic exercise (via cycle ergometer) and an equivalent duration non-exercise control condition. The exercise protocol used here was selected based on evidence showing that the specified duration and intensity provide the largest and most reliable postexercise EF benefit (for meta-analyses see, [17]). For exercise and control conditions, pre- and post-condition oculomotor assessments of EF were completed via the pro- and antisaccade tasks. In line with the ACT, we hypothesized that increased TA would be positively associated with increased antisaccade RTs and that TA would not predict directional errors. As well, we hypothesized a postexercise reduction in antisaccade RTs across the continuum of TA levels reported here, and that a larger magnitude benefit may be observed for individuals with increased TA. The basis for the latter hypothesis stems from work showing that populations with EF deficits exhibit increased reactivity to an exercise intervention (e.g., schizophrenia, autism spectrum disorders) [19], [32].

## 2 Methods

### 2.1 Participants

Thirty-one participants (8 male, 23 female) with an average age of 23 years (SD = 3.7, range: 18–30) from the University of Western Ontario community volunteered for this study. The recruitment for this study involved posters placed on campus community boards and a mass email sent to the University of Western Ontario community. The sample size was determined *a priori* using G*Power (ANOVA: Repeated measures, within factors) with an effect size (*f* = 0.25) derived from a previous study [33] reporting a postexercise EF benefit via the antisaccade task (α = 0.05, power = 0.80).

Inclusion criteria were normal or corrected-to-normal vision; no history of cardiorespiratory disease, neurological/neuropsychiatric disease (including concussion and episodes of major or minor depression); neurodevelopmental disorders; non-smoking; not taking medication for any long-term health issue and/or short-term medication influencing cardiovascular, hemodynamic, or metabolic responses to exercise; and not experiencing an acute illness (i.e., flu-like symptoms). Participants were asked to refrain from consuming alcohol, recreational drugs and partaking in strenuous physical activity 12 h prior to a study session. Participants were also asked to get eight hours of sleep the night before each session. All participants reported adhering to these requirements. Participants were instructed to consume a standard breakfast (e.g., bowl of oatmeal with apple; peanut butter toast and banana) and drink approximately 500 mL of water one hour before a study session. Data collection for all sessions occurred between 9:30 am and 11:00 am in a dedicated laboratory space.

Participants were deemed “physically ready” to exercise via the 2022 Physical Activity Readiness Questionnaire (PAR-Q+) (i.e., answered “No” to the first seven questions) [34]. Participants also completed the Godin Leisure-Time Exercise Questionnaire (GLTEQ) [35] to assess their levels of activity and were required to be “moderately active” (score of 14 or above) to participate. The mean GLTEQ score was 48 (SD = 29; Range = 14–119). Prior to any study procedures, participants read a letter of information and signed a consent form approved by the Health Sciences Research Ethics Board, University of Western Ontario (HSREB #123427). The study was conducted in accordance with the most recent Declaration of Helsinki with the exception that participants were not included in a database.

### 2.2 Experimental overview

**Fig. 1:**
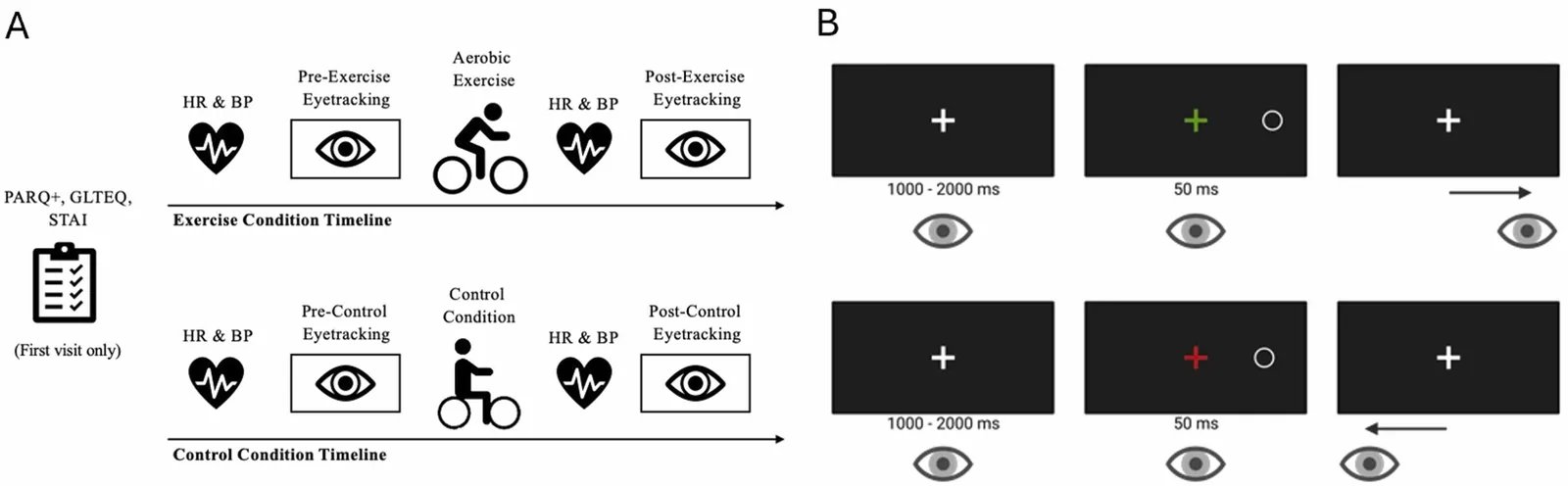
Panel A shows a timeline of experimental events for exercise (top) and control (bottom) conditions. The eye icon represents when oculomotor EF assessments were conducted (i.e., pre- and post-condition). In the exercise condition, following the baseline assessment of HR and BP, an oculomotor assessment was completed (i.e., pre-condition) and participants then completed an exercise intervention followed by a subsequent oculomotor assessment (i.e., post-condition). For the control condition, participants remained seated on the cycle ergometer without pedaling. HR and BP were recorded prior to each intervention (i.e., baseline) and during steady state. Panel B shows the timing and schematic of events for exemplar pro- and antisaccade trials. We note that for ease of presentation only a single target location and eccentricity are presented here.

Participants completed the exercise and control conditions on separate days with each separated by at least 24 h with the order of conditions counterbalanced. On the first visit to the lab, participants read the letter of information, signed the consent form and completed the PAR-Q + and GLETQ. Subsequently, participants completed the STAI and were fitted with heart rate (HR: Polar Electro T34, Kempele, Findland) and automated blood pressure (BP) monitors (Omron Blood Pressure Monitor, Omron Healthcare Inc., Lake Forrest, IL). Following completion of the STAI, HR, and BP were measured to provide a baseline physiological state. The baseline HR and BP measures were completed ∼10 min after participants had arrived at the lab. After baseline, participants completed a “pre-condition” oculomotor EF assessment (see details below) followed by their *a priori* assigned exercise or control condition with HR and BP recorded during the last two minutes of each condition to provide a physiological steady state. Mean arterial pressure (MAP) was then calculated using the formula MAP = (systolic BP + 2(diastolic BP))/3. A subsequent “post-condition” oculomotor EF assessment was completed 5-min following the cessation of a condition (for timeline of experimental events see Fig. 1), and this timing was used because it represents a window in which HR returns to baseline following exercise cessation. On the second visit to the lab, participants completed their assigned condition via the same timeline as described above with the exception that the exercise screening (PAR-Q+ and GLETQ) and STAI were not administered.

For the exercise and control conditions, participants sat on a cycle ergometer (Monark 818E Ergometer, Monark Exercise AB, Vansbro, Sweden) so that their legs achieved approximately 85% of full extension at the end of a pedal stroke. Once positioned, in the exercise condition participants completed a 2.5 min warm-up at 25 watts after which they exercised for 20 min at 64–76% of their maximum predicted HR (i.e., 220 minus age in years; moderate intensity) at a metronome-paced cadence of 70 rpm. During this time, HR and pedal resistance were monitored by the experimenter to ensure a prescribed work rate. A cool-down was initiated after 20 min for the same duration and intensity as the warm-up. For the control condition, participants were seated on the bike for 25 min (time-matched to the exercise condition) without exercising and were able to chat with the experimenters during this time.

### 2.3 State-Trait Anxiety Inventory

Participants completed the TA subscale of the STAI [36] prior to the exercise or control session on their first visit. This 20-item questionnaire uses a 4-point Likert scale ("1 = Almost Never" to "4 = Almost Always") and asks participants to respond to statements such as, “I worry too much about things that don’t really matter” and “I am content; I am a steady person”. Participants rated each statement according to their typical daily feelings. Based on Spielberger et al.’s [36] classification criteria, STAI scores are classified as “no or low anxiety” (20−37), “moderate anxiety” (38−44), and “high anxiety” (45−80).

### 2.4 Oculomotor assessment

Oculomotor EF assessments were completed pre- and post-condition. For each assessment, participants sat on a height-adjustable chair in front of a table on which an LCD monitor (60 Hz, 8-ms response rate, 1280 × 960 pixels; Dell 3007WFP, Round Rock, TX) was located 550 mm from the table's front edge. Participants placed their head on a head/chinrest and the gaze location of their left eye was tracked via a video-based eye tracking system (EyeLink 1000 Plus; SR Research, Ottawa, ON, Canada) sampling at 1000 Hz. Prior to data collection, a nine-point calibration and validation of the viewing space was completed (i.e., <1° of error). All experimental events were controlled via MATLAB (R2018a; The MathWorks, Natick, MA, USA) and the Psychophysics Toolbox extension (v. 3.0) [37], [38] including the EyeLink Toolbox [39]. For all experimental sessions, the lights in the experimental suite produced an ambient luminance of 8 cd/m^2^.

Visual stimuli were presented on a black screen (0.1 cd/m^2^) and included a white fixation cross (1° in diameter: 127 cd/m^2^) and open white target circles (1° in diameter: 127 cd/m^2^). The fixation cross was displayed for a randomized and uniformly distributed foreperiod (1000–2000 ms) after which the target circle was shown for 50 ms 13.5° (proximal target) and 16.5° (distal target) left and right of the fixation and in the same horizontal plane. The fixation cross was presented throughout a trial (i.e., overlap paradigm) and onset of the target served as the imperative to pro- (i.e., saccade to target) or antisaccade (i.e., saccade mirror-symmetrical to target). The brief presentation of the target was used to equate pro- and antisaccade trials for the absence of extraretinal feedback regarding endpoint accuracy. For each oculomotor assessment, pro- and antisaccades were completed in separate and randomly ordered blocks involving 80 trials pseudo-randomly ordered to each target location (i.e., proximal, distal) and visual space (i.e., left and right of fixation). Each oculomotor assessment (including calibration time) required approximately 15 min to complete.

### 2.5 Data reduction, dependent variables, and statistical analyses

For the oculomotor task, gaze position data were filtered offline using a dual-pass Butterworth filter employing a low-pass cut-off frequency of 15 Hz. A five-point central-finite difference algorithm was used to compute instantaneous velocities and accelerations. Saccade onset was determined when velocity and acceleration exceeded 30°/s and 8000°/s^2^, respectively. Saccade offset was determined when velocity fell below 30°/s for 40 ms. Trials involving signal loss (e.g., an eye blink) were removed, as were anticipatory responses (RTs < 50 ms) [40] and RTs > 2.5 standard deviations of a participant- and task-specific mean [14]. Trials involving a directional error (i.e., a prosaccade instead of an instructed antisaccade and *vice versa*) were excluded from RT, saccade duration, and gain variability analyses because they are associated with planning mechanisms distinct from their directionally correct counterparts [41].

Physiological dependent variables included HR and MAP. Oculomotor dependent variables included RT (i.e., time from response cueing to saccade onset), percentage of directional errors, saccade duration (i.e., time from saccade onset to saccade offset), and saccade gain variability (i.e., within-participant standard deviation of saccade amplitude/veridical target location). All data met the assumption of normality (Q-Q plots and Shaprio-Wilk test) and were examined via repeated measures linear mixed-effect models. For HR and MAP, the model included **condition** (control, exercise) and **time** (baseline, steady state) as fixed effects, whereas for the oculomotor data the model included **condition** (control, exercise), **time** (pre-, post-condition) and **task** (pro-, antisaccade) as fixed effects. For all models, participant was entered as a random intercept and baseline STAI scores were grand-mean centered and entered as a between-participant covariate to control for initial individual differences. Linear mixed-effects models were computed via R (Version 4.6.0) using the lme4 package (Version 2.0.2; [42]), with parameters estimated via Restricted Maximum Likelihood (REML) and *p*-values and degrees of freedom derived via the lmerTest package (Version 3.2.1; [43]) and Satterthwaite’s method for approximating degrees of freedom. In addition, and where appropriate, frequentist and Bayesian *t*-tests were used. Bayesian *t*-tests were used to evaluate the null hypothesis (i.e., BF_01_). Jeffreys’ [44] nomenclature of “anecdotal” (i.e., 1 to <3), “moderate” (i.e., 3 to <10), “strong” (i.e., 10 to <100) and “very strong” (i.e., >100) was used to contextualize Bayes factor robustness.

## 3 Results

STAI scores ranged from 23 to 60 and produced an average and median of 42 (SD = 10, SIQR = 7.75). The range of scores is comparable to published norms for male (38, SD=10) and female (40, SD = 12) participants of comparable age and education levels [36].

### 3.1 Heart rate and mean arterial pressure

**Fig. 2:**
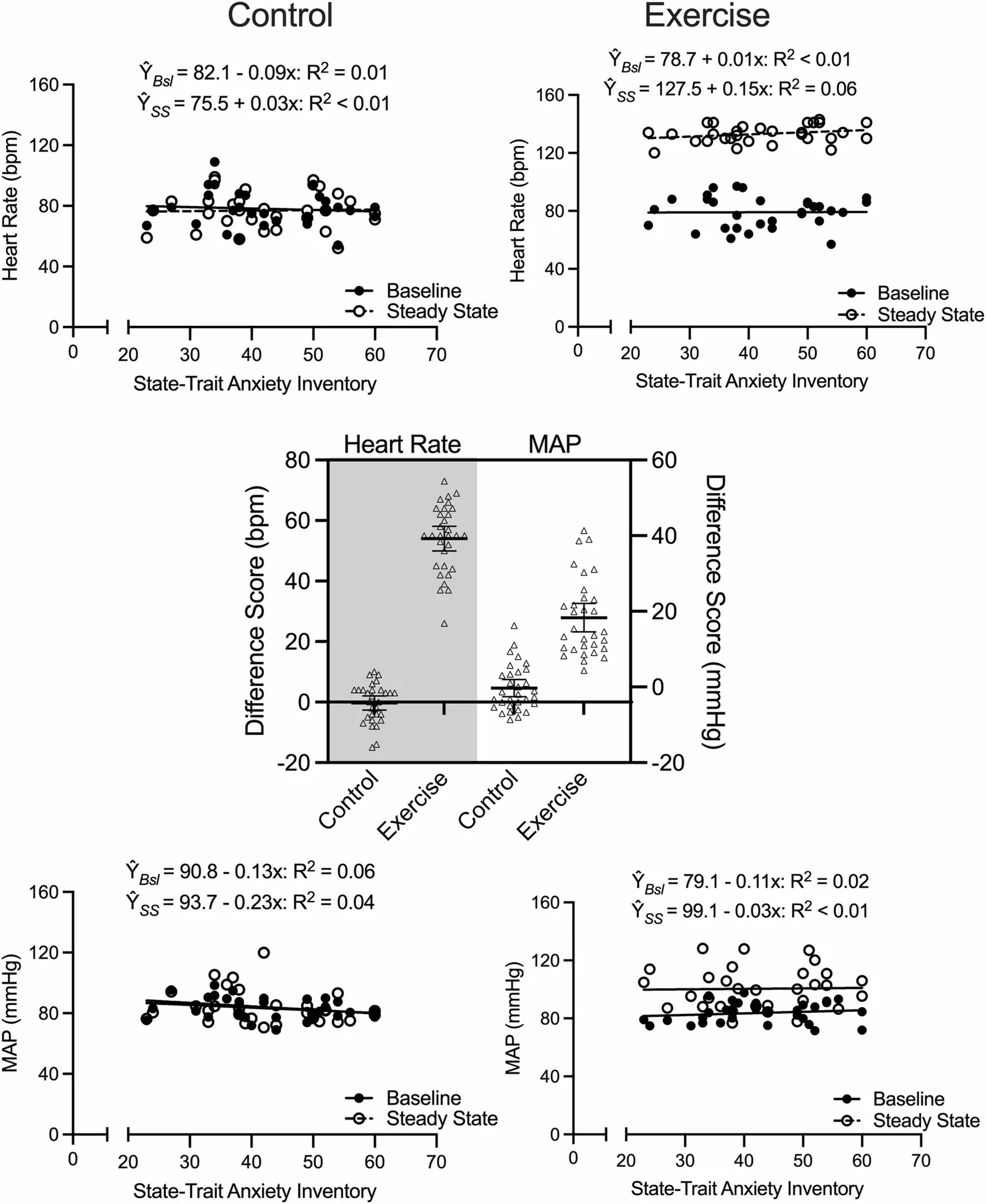
Scatterplots of participant-specific State-Trait Anxiety Inventory values as a function of baseline (B_sl_) and steady state (SS) heart rate (top panels, in beats/min [bpm]) and mean arterial pressure (bottom panel, MAP in mmHg) separately for control (left panels) and exercise (right panel) conditions. For each panel, linear regression equations and proportion of explained variance are provided separately for baseline and steady state. The central panel provides participant-specific and group mean heart rate and MAP difference scores (steady state minus baseline) for control and exercise conditions. Error bars represent 95% between-participant confidence intervals and the absence of overlap between an error bar and zero represents a reliable difference inclusive to a test of the null hypothesis.

STAI was not a significant predictor of HR, *b* = 0.02, *SE* = 0.16, *t*(29) = 0.14, *p* = .889, 95% CI [−0.30, 0.34] or MAP, *b* = −0.15, *SE* = 0.12, *t*(29) = −1.20, *p* = .242, 95% CI [−0.40, 0.11]. In turn, results yielded **condition** by **time** interactions for HR, *b* = 55.16, *SE* = 2.72, *t*(30) = 20.31, *p* < .001, 95% CI [49.84, 60.48], and MAP, *b* = 17.39, *SE* = 3.41, *t*(30) = 5.10, *p* < .001, 95% CI [10.43, 24.36]. For the control condition, neither HR, *b* = −1.10, *SE* = 1.92, *t*(30) = −0.57, *p* = .572, 95% CI [−4.86, 2.67], nor MAP, *b* = 0.66, *SE* = 2.41, *t*(30) = 0.27, *p* = .788, 95% CI [−4.27, 5.58], produced a reliable difference between baseline (HR = 78 bpm, SD = 12; MAP = 93 mmHg, SD = 7) and steady-state (HR = 76 bpm, SD = 11; MAP = 84 mmHg, SD = 11), whereas for the exercise condition HR, *b* = 54.06, *SE* = 1.92, *t*(30) = 28.15, *p* < .001, 95% CI [50.30, 57.82], and MAP, *b* = 18.05, *SE* = 2.41, *t*(30) = 7.49, *p* < .001, 95% CI [13.12, 22.98], increased from baseline (HR = 79 bpm, SD = 11; MAP = 83 mmHg, SD = 7) to steady state (HR = 133 bpm, SD = 6; MAP = 102 mmHg, SD = 15) (Fig. 2).

### 3.2 Oculomotor assessment

#### 3.2.1 Reaction time

**Fig. 3:**
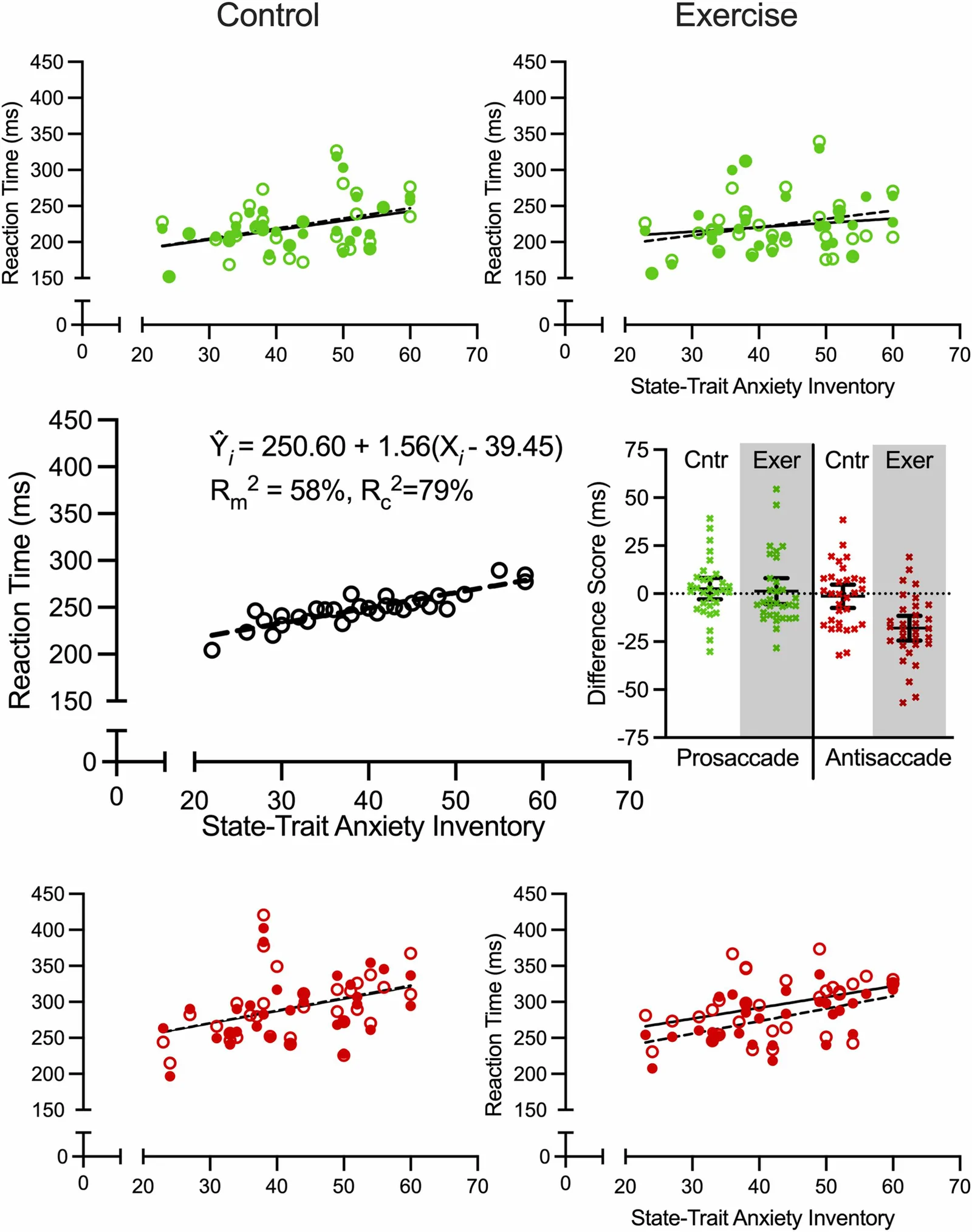
The scatterplots provided in each corner of the figure represent participant-specific State-Trait Anxiety Inventory values as a function of control (left panels) and exercise (right panels) condition pre- (open symbols) and post-condition (closed symbols) pro- (top panels and green symbols) and antisaccade (bottom panels and red symbols) reaction times. In the larger central scatterplot, participant-specific State-Trait Anxiety Inventory values are plotted as a function of grand mean reaction times (i.e., collapsed across time and task) with the regression line representing the fixed-effect regression slope predicted by the linear mixed model. The regression equation for the grand mean outcomes as well as the marginal (R_m_^2^) and conditional (R_c_^2^) proportions of explained variance is presented at the top of the panel. The figure adjacent to the central panel provides participant-specific and grand mean pro- and antisaccade reaction time difference scores (post-condition minus pre-condition) separately for control (Cntr) and exercise (Exer) conditions. Error bars in the figure represents the 95% between-participant confidence interval and the absence of overlap between an error bar and zero represents a reliable difference inclusive to a test of the null hypothesis. Notably, this panel directly demonstrates that only exercise condition antisaccades difference scores decreased from pre- to post-condition assessment.

Fig. 3 shows that the STAI covariate in our model was significant, *b* = 1.38, *SE* = 0.59, *t*(29) = 2.33, *p* = .027, 95% CI [0.22, 2.54], such that higher STAI values predicted longer RTs across all experimental conditions. As well, results revealed a main effect of **task**, *b* = 69.25, *SE* = 5.83, *t*(30) = 11.87, *p* < .001, 95% CI [57.82, 80.69], and interactions involving **condition** by **task**, *b* = -4.28, *SE* = 6.25, *t(30)* = 2.46, *p* = .02, 95% CI [-14.89, −5.46,], and **condition** by **task** by t**ime**, *b* = -15.31, *SE* = 5.43, *t*(30) = 2.22, *p* ≈ 0.034, 95% CI [-18.89, −8.18]. The main effect of **task** revealed the expected finding that RTs were shorter for pro- (212 ms, SD = 51) than antisaccades (289 ms, SD = 43). In decomposing the three-way interaction, we examined the effect of **task** and **time** separately for control and exercise conditions via simple effects. As shown in the corner panels of Fig. 3, control condition pro- and antisaccade RTs did not differ from pre- to post-condition, *b* = -3.96, *SE* = 8.55, *t*(30) = −0.46, *p* = .643, 95% CI [−20.72, 12.80], whereas for the exercise condition a **time** by **task** interaction, *b* = −19.27, *SE* = 6.48, *t*(30) = −2.98, *p* = .003, 95% CI [−20.96, −6.58] indicating that antisaccade – but not prosaccade – RTs decreased from pre- to post-intervention. As well, Fig. 3 provides participant-specific and grand mean RT difference scores (i.e., pre-condition minus post-condition) and associated 95% confidence intervals separately for control and exercise conditions. This figure demonstrates that the exercise condition produced a selective decrease in antisaccade RT difference scores.

Given the primary objective of this study, in addition to the linear mixed-effect analysis, we performed a median split based on STAI values to stratify participants into low- and high-TA groups. This is a statistical approach directly matching previous work examining TA-related EF differences [6], [7], [16]. The median split value of 42 resulted in the low-TA group being composed of participants meeting the classification of “low-to-no” (N = 10) and “moderate” (N = 5) anxiety, whereas the high-TA group was composed of individuals with the classification of “moderate**”** (N = 4) to “high” (N = 12) TA (see Table 1 for group demographics). For this median split, we examined whether the magnitude of a postexercise antisaccade RT reduction differed between low- and high-TA groups. Accordingly, we computed antisaccade RT difference scores (i.e., pre-condition minus post-condition) and employed frequentist independent samples *t*-tests and a Bayesian test of the null hypothesis (i.e., BF_01_). The frequentist analysis indicated that antisaccade RT difference scores did not reliably differ between low- and high-TA groups, *t*(29) = -0.97, p = 0.34, d_z_ = -0.35) and the Bayesian analysis (BF_01_ = 2.06) provided anecdotal support for a null between-group difference. In other words, low- and high-TA groups exhibited a comparable magnitude pre- to postexercise reduction in antisaccade RTs.

**Table 1:** Participant demographics.

Variable	Mean	
	Low TA (n = 15)	High TA (n = 16)
Age	24	22
Biogical sex (M/F)	6/10	3/13
GLTEQ Score	52 (SD = 32)	46 (SD = 29)
STAI_range_	23–40	42–60
STAI_x̅_	34 (SD = 5)	51 (SD = 6)
cHR_Baseline_ (bpm)	80 (SD = 14)	76 (SD = 10)
cHR_Steady state_ (bpm)	78 (SD = 13)	76 (SD = 13)
eHR_Baseline_ (bpm)	80 (SD = 13)	79 (SD = 9)
eHR_Steady state_ (bpm)	132 (SD = 6)	135 (SD = 6)
cMAP_Baseline_ (mmHg)	85 (SD = 8)	82 (SD = 6)
cMAP_Steady state_ (mmHg)	86 (SD = 11)	82 (SD = 12)
eMAP_Baseline_ (mmHg)	84 (SD = 8)	83 (SD = 7)
eMAP_Steady state_ (mmHg)	101 (SD = 13)	102 (SD = 17)

*Note.* TA = trait anxiety; cHR = heart rate during control condition; eHR = heart rate during exercise condition; cMAP = mean arterial blood pressure during control condition; eMAP = mean arterial blood pressure during exercise condition; GLTEQ = Godin Leisure-Time Exercise Questionnaire

#### 3.2.2 Directional errors

STAI values did not predict directional errors, *b* = 0.04, *SE* = 0.15, *t*(29) = 0.24, *p* = .811, 95% CI [−0.25, 0.32]; however, results produced main effects for **time**, *b* = −4.66, *SE* = 2.33, *t*(30) = −2.00, *p* = .046, 95% CI [−9.23, −0.09], and **task**, *b* = 7.16, *SE* = 2.33, *t*(30) = 3.07, *p* = .002, 95% CI [2.60, 11.73]. Directional errors decreased from pre- (11%, SD = 11) to post-condition (7%, SD = 6) and were fewer for pro- (5%, SD = 6) than antisaccades (12%, SD = 10). Neither the main effect of **condition** nor any higher order interaction involving **condition** were significant, *t*s < |1.82|, *p*s > .09.

#### 3.2.3 Saccade duration and gain variability

STAI did not predict saccade duration, *b* = −0.08, *SE* = 0.18, *t*(29) = −0.44, *p* = .661, 95% CI [−0.44, 0.28], or gain variability, *b* = 0.001, *SE* = 0.001, *t*(29) = 1.14, *p* = .253. In turn, results yielded main effects of **task** for saccade duration, *b* = 7.21, *SE* = 2.25, *t*(30) = 3.20, *p* = .003, 95% CI [2.80, 11.63], and gain variability, *b* = 0.094, *SE* = 0.011, *t*(30) = 8.39, *p* < .001, 95% CI [0.072, 0.116]. Prosaccades had shorter durations (56 ms, SD = 11) and less variable endpoints (0.13, SD = 0.05) than antisaccades (saccade duration: 64 ms, SD = 15; gain variability: 0.23, SD = 0.07). The main effects for **condition** as well as higher-order interactions involving **condition** were not significant for either saccade duration or gain variability, *t*s < 0.92, *p*s > .35).

## 4 Discussion

This study sought to determine whether TA levels influence baseline differences in an oculomotor assessment of EF and whether a single bout of aerobic exercise benefits EF across a continuum of TA. In discussing our findings, we first outline the general differences between pro- and antisaccade metrics and baseline EF in the low- and high-TA groups before outlining the impact of the exercise intervention.

### 4.1 Antisaccade performance metrics evince top-down executive function

A general finding from this study was that antisaccades produced longer RTs and saccade durations as well as increased directional errors and endpoint variability compared to prosaccades. The prosaccade metrics demonstrate that an oculomotor response with standard stimulus-response mapping is mediated largely independent of top-down EF via direct retinotopic projections to the superior colliculus [10]. In contrast, the longer RTs for antisaccades reflect the time-consuming EF demands of suppressing a reflexive prosaccade (i.e., inhibitory control) and performing a 180° spatial transposition of a target’s coordinates (vector inversion) (for review see [15]). In turn, increased antisaccade directional errors have been shown to reflect the inability to evoke the high-level ‘task-rule’ or ‘task-set’ associated with an EF task [45]. Furthermore, that antisaccades produced longer saccade durations and increased endpoint variability has been linked to: (1) visuomotor uncertainty stemming from decoupling stimulus-response spatial relations [46] and, (2) response mediation via visual information (i.e., relative) functionally distinct from the metrically precise visual information supporting prosaccades [47], [48], [49], [50]. Thus, the distinct pro- and antisaccade metrics observed here provide a basis for investigating whether low- and high-TA groups exhibit distinct baseline and postexercise changes in an oculomotor index of EF.

### 4.2 Baseline oculomotor control in low- and high-TA groups

STAI values showed a positive relationship with baseline (i.e., pre-condition) and post-condition pro- and antisaccade RTs and the estimated increase in pre-condition antisaccade RTs from the lowest to highest STAI value in our sample (i.e., 23–60) was 50 ms (SD = 22). The RT increase broadly aligns with Ansari and Derakshan’s [6] findings showing that antisaccade RTs for their low-TA group (STAI < 34) were 27 ms shorter than their high-TA group (STAI > 44). As well, and in line with Ansari and Derakshan, our finding showed that STAI did not influence antisaccade directional errors. Thus, the selective examination of baseline antisaccade metrics supports the ACT’s assertion that increased TA renders decreased EF efficiency (i.e., RT) without a change in effectiveness. (i.e., directional errors). It is, however, important to recognize that we found that STAI values showed a positive linear relationship with prosaccade RTs – a result differing from Ansari and Derakshan who reported a null difference between their low- and high-TA groups. Accordingly, the present findings lend to the explanation that increased TA elicits a general increase in information processing time and not a selective EF deficit. That said, an important difference between our work and Ansari and Derakshan is that we used an overlap saccade paradigm (i.e., fixation present at target onset), whereas Ansari and Derakshan employed a gap paradigm (i.e., fixation extinguished 200 ms prior to target onset). Notably, the concurrent presentation of a fixation location at the time of target onset renders increased competition between fixation and burst neurons within the superior colliculus [51] that may induce inhibitory control across pro- and antisaccade trials. In contrast, fixation neuron activity actively dissipates during a gap interval and may decrease top-down inhibitory control for prosaccade planning. Of course, such an explanation requires a purpose-designed study to address whether TA-status impacts baseline pro- and antisaccade RTs across gap and overlap paradigms. A second distinction between the current work and Ansari and Derakshan, as well as other work examining EF in “low” and “high” TA [16], is that participants in the present study met a “recreational” level of physical activity as determined via the GLTEQ, whereas previous work did not quantify physical activity levels. Given evidence that adherence to regular physical activity improves EF (for review see, [52]) and that increased TA has been reported as a salient barrier to adherence to regular physical activity [53], [54], [55] it is possible that physical activity and/or physical fitness levels may – in part – influence between-group differences in baseline EF. Thus, we believe the present findings add importantly to the literature insomuch as they demonstrate that TA status results in an increase in pro- and antisaccade RTs across participants with comparable levels of recreational activity and identifies that recreational and/or cardiovascular fitness should be considered as potential moderators in evaluating the impact of TA on EF (see also [17]).

### 4.3 Exercise improved EF across the continuum of STAI values

The results of our linear mixed model indicated that the exercise condition produced a null pre- to post- change in prosaccade RTs – a finding accounted for by evidence showing that the pre-potent nature of prosaccades [10] does not result in a general exercise-based improvement in information processing [17]. In contrast, a pre- to postexercise reduction was observed for antisaccade RTs, and as shown in Fig. 3, was a result consistent across the continuum of STAI values reported here. As well, in a secondary analysis involving the stratification of participants based on STAI values, frequentist and Bayesian statistics showed that the magnitude of the postexercise antisaccade RT reduction did not differ between low- and high-TA groups. The exercise-based reduction in antisaccade RTs is unlikely related to a practice-related performance benefit given that the control condition did not elicit RT or directional error changes. Moreover, pro- and antisaccade duration and gain variability – across exercise and control conditions – did not vary across assessment timepoints. As a result, the selective reduction in antisaccade RTs in the exercise condition cannot be attributed to an implicit – or explicit – control strategy designed to decrease RT at the cost of decreased endpoint accuracy (i.e., speed-accuracy trade-off) [56]. Instead, the results support a number of studies by our group [21], [22], [25], [33], [57] and others [58], [59] reporting that antisaccade – but not prosaccade – planning mechanisms elicit a postexercise benefit (for extensive review see, [60]). What is more, our findings accord a more expansive literature indicating that a single bout of exercise benefits EF (for meta-analyses see, [17], [18], [20], [60], [61]. Further, we note that our measured physiological variables (HR and MAP) in the exercise condition showed the expected baseline to steady state change for a moderate intensity exercise intervention and these changes were unrelated to STAI values.

To our knowledge, only two previous studies examined the impact of exercise on TA. In one study, Ligeza et al. investigated the impact of a single 20 min bout of aerobic exercise at 65–75% of HR_max_ (i.e., 220 minus age in years) on EF (via modified Flanker task) in preadolescents (ages 9–11 years) with low and high-TA (via the STAI for Children [STAIC]) [62]. Results showed that the exercise intervention did not improve EF for either group. This represents an unexpected finding given that the exercise neuroscience/psychology literature has provided a general consensus that a single bout of exercise benefits EF in children (for meta-analyses see, [17], [18]). In a second study, Dong et al. [53] classified young adults as having low-, medium-, or high-TA and measured their self-reported levels of physical activity (International Physical Activity Questionnaire) and EF performance (i.e., Stroop, 1-back and set-shifting). Results showed that physical activity levels and EF were positively associated and thus underscores the moderating link between physical activity level with TA status and EF. Importantly, that tour findings demonstrated a post-exercise reduction in antisaccade RTs demonstrates that individuals can accrue a postexercise EF benefit regardless of their TA status.

### 4.4 Study limitations

We acknowledge that our results are limited by several methodological constraints. First, we did not include a measure of state anxiety in conjunction with TA. We elected to not include this measure because the literature offers mixed findings on the relationship between state anxiety and TA, and because they are thought to be supported by distinct structural and functional brain mechanisms [63]. In addition, our goal was to examine postexercise EF benefits across individuals with a stable personality dimension of TA. That said, future work could assess how TA combined with a current state anxiety influence a postexercise EF benefit. Second, our work examined only healthy young adults, and it is therefore unclear whether exercise-related EF benefits would similarly characterize older adults with comparable levels of TA or individuals experiencing a chronic disease. Third, our study employed a single exercise intensity and duration. As a result, we are unable to assert whether longer and more intense exercise interventions would differentially impact postexercise EF benefits across the continuum of TA status. Fourth, although the inclusion criteria specified that participants reported no history of neurological or neuropsychiatric disease and were not taking medication for any chronic medical condition, we did not quantify whether factors such as early life experiences, socioeconomic status, social functioning, genetics, and/or individual-specific personality traits may have influenced baseline or postexercise oculomotor control [4], [5], [64]. Such factors should be considered in future work and employ a larger sample size in determining whether underlying TA-specific factors influence postexercise EF benefits. Last, we did not include concurrent neurophysiological (i.e., transcranial Doppler ultrasound) or neuroimaging (i.e., EEG, fNIRS) measures for our EF task. As such, and although individuals exhibited comparable behavioural metrics independent of TA status, it may have been accomplished via distinct task-based activity within EF networks. Despite these limitations, we note that to our knowledge, our work provides first evidence that a single bout of aerobic exercise benefits EF across a continuum of TA.

## 5 Conclusions

STAI values were positively associated with pro- and antisaccade RTs at baseline and postexercise assessments. More notably, our exercise intervention yielded a selective reduction in antisaccade (but not prosaccade) RTs without an associated change in directional errors across the continuum of STAI values. Thus, results underscore the important link between exercise and EF and provide a roadmap for future research to understand how participant-specific exercise interventions (i.e., duration and intensity) may support improved EF for individuals across the spectrum of TA.

## Author Contributions

LB and MH conceived and designed the research, LB, GJ and AR performed experiments; LB, MH and BT analyzed data; LB, MH and BT interpreted results of experiments; LB, MH and BT prepared figures; LB, MH and BT drafted the manuscript; LB, GJ, AR, MH and BT edited and revised the manuscript; LB, GJ, AR, MH and BT approved the final version of the manuscript.

## Ethics Approval

This work was approved by the Health Sciences Research Ethics Board, University of Western Ontario (HSREB #123427) and was conducted according to the most recent iteration of the Declaration of Helsinki.

## Funding

This work is supported by a Discovery Grant (MH) from the Natural Sciences and Engineering Research Council (10.13039/501100000038NSERC) of Canada, and Faculty Scholar and Major Academic Development Fund Awards from the 10.13039/501100004381University of Western Ontario.

## Declaration of Competing Interest

The authors have no competing interests to declare that are relevant to the content of this article.

## Data Availability

Data will be made available upon reasonable request.
